# Supplementary material for: Barriers to conducting independent quantitative research in low-income countries: A cross-sectional study of public health graduate students in Liberia
Source: PLoS One. 2023 Feb 2;18(2):e0280917. doi: 10.1371/journal.pone.0280917 (PMC9894428; doi:10.1371/journal.pone.0280917)
Supplement: Attachment — Submitted filename: Response to Reviewers.docx [file pone.0280917.s003.docx]

December 22, 2022

Dear Dr. Mohan Kumar:

We wish to thank the reviewers for the insightful and helpful comments on our manuscript. We are thus pleased to submit a revised version of the paper, “Barriers to conducting independent quantitative research in low-income countries: a cross-sectional study of public health graduate students in Liberia” as invited by the decision letter for PONE-D-22-27103. We have modified the manuscript to address the comments and suggestions of the reviewers as detailed below. Please find each of the reviewers’ comments in *italics* and our responses in normal face.

Thank you for your consideration of our revised manuscript. We look forward to hearing from you.

Sincerely,

Hajah Kenneh, MPH

*Reviewer #1:*

*1. The authors have submitted a well-written manuscript including ethical aspects. Methods, results, discussion, and conclusion are detailed and concise. The manuscript is acceptable in the current form for publication.*

Thank you for the thoughtful feedback. We are eager to share the work and indeed hope that the results guide stakeholders interested in building research capacity in Liberia and other similar contexts.

*Reviewer #2:*

*1. This study is potentially interesting, but its relatively simple design (cross sectional) and small sample limit the interest of the study. I think that a qualitative study (focus group or interview) would have provided more interesting information than a quantitative study.*

Thank you for this comment. We expect that our findings will form the basis of ongoing research into barriers so that research capacity-building efforts are more informed. Larger studies with mixed method designs would indeed strengthen the evidence provided in our baseline investigation. We have commented on the need for further research and the value of qualitative data to provide more nuanced findings in the Limitations section of the Discussion.

*2. Abstract: It is necessary to add in this results section some aOR with their 95% CI, even if they are not significant.*

We appreciate the suggestion and have updated the last sentence of the Results section in the Abstract to include the relevant aOR and 95% CI. The sentence now states: “Computer ownership was found to be statistically significantly associated with higher likelihood of conducting quantitative research in the multivariable analysis (aOR: 4.90, 95% CI: 1.54 - 16.3).”

*3. Introduction: This introduction section should be more developed so that the reader understands the context, the issue and the interest of this study.*

The feedback on the Introduction section is appreciated. We have added a paragraph to provide more information about the research context in Liberia, and the interest of the study to provide understanding about the context for those interested in building research capacity, and/or designing investments to do so, in Liberia. Wehave likewise updated the final paragraph to explain our objective more explicitly.

*4. Introduction: In addition, the objective of the study should be clearly defined at the end of this section.*

*W*e have added more clarity on the objective of the study at the end of the Introduction Section, which now includes the following statement: “The study aims to provide evidence around resource needs to enable tertiary- and graduate-level capacity for quantitative sciences in settings like Liberia with limited national resources for education and technology.”

*5. Methods: How did you choose the questions for the questionnaire? did you take them from other existing studies? or did you develop them specifically for this study?*

Thank you for the comment. The selection of the questions for the questionnaire was two-fold. Approximately 20% of the questions in the questionnaire were adapted and revised from an existing study (Peter AM, 2017) while 80% were developed by the researchers. Within the Data Collection Tool section of the Methods, we have added this sentence: “The majority of questions on the tool was based on the researchers’ experience teaching and learning in Libera; approximately 5-10 questions (20%) on the tool were adapted from a survey previously used [21].”

*6. Methods: They were tested on a small group of students before being sent to the participants? More information should be given to the reader for the replication of the study*

The questionnaire was pretested on a small group of students from the Medical School and R Beginner students at Quantitative Data for Decision-Making Lab (Q4D-Lab). The pretest was intended to demonstrate participants' understanding of the questionnaire and to provide a measure of the time required for completion. The text included the number of persons completing the pretest and the action taken based on our observations during the process. However, to provide further detail, we have now added the following: “We documented on average how long it took the students to complete the tool and whether responses were consistent with expectation,” in the Data Collection Tool section of the Methods.

*7. Tables: in table 5 if you have the 95% CI you don't need the p value*

*The point is well taken. P-values have been removed from the Table.*
